# Prevalence and risk factors for modified prescriptions in an Irish community pharmacy

**DOI:** 10.1186/1753-6561-9-S1-A48

**Published:** 2015-01-14

**Authors:** S Obasi, A Tinsley, F Doyle

**Affiliations:** 1 Division of Population Sciences (Psychology), Royal College of Surgeons Ireland, 123 St. Stephen’s Green, Dublin 2. Ireland; 2 McCabes Pharmacy, Dublin, Ireland

## Background

Little research exists of rates of prescribing errors and prescription modifications, or risk factors for same, in Ireland.

## Methods

A cross-sectional study was performed to examine prescriptions dispensed in a community pharmacy over a period of 5 weeks from November 19 to December 21 2012.

## Results

In total, 866 prescriptions were examined. The overall prevalence of prescription modifications was 17.9% (155/866), with a mean of 31 modifications per week. Prescription only medicines (POM) comprised 147 (94.8%) of the modifications, 128 (87.1%) of which concerned a prescription error requiring a simple clerical clarification before dispensing could occur, with the remaining 19 (12.9%; average of 3.8 per week) potentially having clinical consequences if left unaltered. Half (51%) of all POM modifications occurred through consultation with the patient or their representative. The following factors were associated with increased risk of POM modifications: being a female patient (OR = 1.605, 95% CI 1.104-2.333, p = 0.013) and being prescribed drugs in the following therapeutic areas: musculo-skeletal (OR = 1.906, 95% CI 1.023-3.551, p = 0.042) and genito urinary system and sex hormones (OR = 3.691, 95% CI 2.255-6.042, p< 0.001). Multivariate analysis showed these were significant independent determinants of POM modifications, remaining so after adjustment.

## Conclusions

The majority of prescribing errors modified involved non-serious clerical errors. However an average of 3.8 POM prescriptions with potential clinical consequences were modified weekly.

